# Latest Trends in Lipase-Catalyzed Synthesis of Ester Carbohydrate Surfactants: From Key Parameters to Opportunities and Future Development

**DOI:** 10.3390/ijms25073727

**Published:** 2024-03-27

**Authors:** Alexis Spalletta, Nicolas Joly, Patrick Martin

**Affiliations:** Unité Transformations & Agroressources, ULR7519, Université d’Artois-UniLaSalle, F-62408 Béthune, France; alexis.spalletta@univ-artois.fr (A.S.); patrick.martin@univ-artois.fr (P.M.)

**Keywords:** sugar fatty acid ester, biocatalysis, lipase

## Abstract

Carbohydrate-based surfactants are amphiphilic compounds containing hydrophilic moieties linked to hydrophobic aglycones. More specifically, carbohydrate esters are biosourced and biocompatible surfactants derived from inexpensive renewable raw materials (sugars and fatty acids). Their unique properties allow them to be used in various areas, such as the cosmetic, food, and medicine industries. These multi-applications have created a worldwide market for biobased surfactants and consequently expectations for their production. Biobased surfactants can be obtained from various processes, such as chemical synthesis or microorganism culture and surfactant purification. In accordance with the need for more sustainable and greener processes, the synthesis of these molecules by enzymatic pathways is an opportunity. This work presents a state-of-the-art lipase action mode, with a focus on the active sites of these proteins, and then on four essential parameters for optimizing the reaction: type of lipase, reaction medium, temperature, and ratio of substrates. Finally, this review discusses the latest trends and recent developments, showing the unlimited potential for optimization of such enzymatic syntheses.

## 1. Introduction

Surfactants play diverse and significant roles, including in the petroleum, soap, and detergent industries; environmental depollution; and even the food industry [1,2]. For example, they possess the ability to reduce air–water and oil–water interfacial tension [3]. They have gained attention because of their diverse and extensive applications. This set of molecules is mainly obtained through chemical processes, with a significant impact on the environment, or microbiological means, as with rhamnolipids. Biosurfactants, which are surfactants directly extracted from microorganisms, have various structures and functions, in addition to their biodegradability [4,5,6]. They are produced under variable and atypical conditions, requiring appropriate culture media [7]. Biosurfactants are applicable in agriculture [3], in the food industry [8], in biomedicine [9], in nanotechnology [10], and in other diverse fields, including detergents [11]. In the food industry, surfactants can be used as bio-emulsifiers and preservatives [12]. At the agricultural level, surfactants are described as acting against phytopathogenic fungi or as seed fertility enhancers, or as antimicrobial agents, like 6′-*O*-lactose esters [13,14,15]. In the literature, in biosurfactants based on carbohydrate platforms, either mono- or oligosaccharides are found, and lipids are major constituents. Polysaccharides, amino acids, and peptides are also listed [16]. Carbohydrate esters, classically described as sugar fatty acid esters (SFAEs), are an example of amphiphilic molecules based on carbohydrates. However, production of carbohydrate surfactant by microorganisms, among which the most famous are rhamnolipids, is limited by many factors, such as long purification processes and high production costs [17]. The alternative, especially in terms of synthesis cost, would be to turn to so-called classical chemistry syntheses. Nevertheless, classical chemistry is difficult to implement with carbohydrates, due to their poly-hydroxylated nature [18,19]. The production of surfactants generates a non-negligible number of secondary products [20]. This is therefore a major issue; it is necessary to have new ways to design surfactants that are green and from renewable resources [21]. There is an urgent need for more sustainable industrial processes in line with the principles of green chemistry [22]. Therefore, manufacturers and academics are studying alternatives to chemical surfactants. Indeed, the scientific community is increasingly concerned about their environmental impacts, especially their poor ability to be easily degrade in the environment. Various and numerous studies have been conducted over roughly the past thirty years to find alternatives to synthetic surfactants, with similar properties but environmentally friendly [23,24]. A part of the answer is fatty acid esters and carbohydrate esters, which are non-ionic surfactants, like sucrose or glucose esters, structurally close to glycolipid biosurfactants produced by microorganisms and obtained from naturally occurring renewable resources [25,26]. 

**Figure 1:**
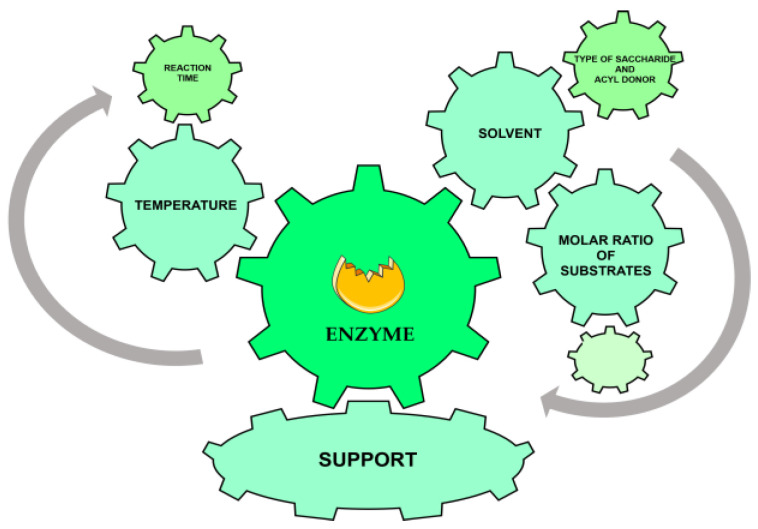
Schematic representation of some optimizable parameters for enzymatic biocatalysis by lipase. All parameters are linked or influence each other.

Biocatalysis appears to be a possible solution for designing biosourced molecules, with enzymes as biocatalysts, to overcome many barriers, according to green chemistry principles. Over the past two decades, interest in biocatalytic transformations has grown. This is partly due to advances in genomic sequencing and bioinformatics, which have made it possible to identify numerous enzymes that are now commercially available [27]. This has facilitated the large integration of biocatalysis, as a mature sustainable technology, into traditional (industrial) organic synthesis, for the enantiospecific synthesis of carbohydrate-derived surfactants [28]. Enzymatic engineering selectively allows for the production of monodispersed molecules with high added value [29]. The attractiveness of enzymes is due to their unique properties as catalysts: high specificity, high selectivity, and limited post-synthesis processing steps. The mild conditions allowed by enzymes, and thus the resulting resource savings, make biocatalysis attractive. Enzymes have great versatility, as their active sites can convert different substrates under varying conditions of temperature and alternative solvents. The demonstration that certain enzymes, especially lipases, can catalyze the conversion of hydrophobic compounds in non-aqueous solvents has stimulated research on the use of biocatalysis in synthesis [30]. Nowadays, enzymes have become affordable, even for large-scale applications. The enzymatic portion of global synthetic product cost is low [31]. Enzymes have become the most relevant biocatalysts for various applications [32], especially because they are able to catalyze both hydrolysis and esterification reactions. Over the last 30 years, there has been a steady increase in the number of publications related to the use of enzymes [33], and it is a field that is still booming [34]. The number of biocatalysis patents is also increasing [35]. Optimization of biocatalyzed reactions requires the consideration of numerous factors, such as catalytic activity parameters, substrate availability, and process economics (Figure 1). 

This review focuses on the recent advances of the last ten years concerning the synthesis of ester surfactants and carbohydrate ester surfactants by lipase into molecules safe for nature and the environment [20]. A specific focus is placed on the enzymatic synthesis and optimization of SFAEs. This work focuses on the different parameters that can be optimized and on the latest advances in the field of biocatalysis by lipase. The second scope of this work will be to discuss improvements to and opportunities for enzymatic catalysis in the 21st century, with a special focus on the trans-disciplinarity of biocatalysis.

## 2. Optimization of Lipase Enzymatic Carbohydrate Ester Synthesis in the 21st Century: Influence of Key Parameters

The kinetics of SFAE lipase-catalyzed reactions are governed by several factors, such as reaction temperature, stirring speed, reagent–enzyme ratio, and the reaction solvent used. The optimization of such syntheses implies the need to study and optimize these parameters either to improve yields, influence enantioselectivity or chemoselectivity, or simply decrease the global carbon footprint of the final product. 

### 2.1. Enzyme Selection

First, it is useful to look at the main actor of these enzymatic ester reactions: lipase catalyst. In the Enzyme Commission nomenclature (EC), lipases are hydrolases, with a three-dimensional structure described as a clamp structure, or sandwich structure, with parallel and antiparallel chains. Indeed, they are constituted by a hydrophobic core region composed of a 7-stranded β-sheet of which 6 are parallel and the 7th is antiparallel. These sheets are surrounded by 10 α-helices. More precisely, lipases are specific carboxylesterases, specifically characterized by the distribution of hydrophobic amino acids in the active site neighborhood [36]. The active site of lipases consists of the following amino acid motif: Ser-His-Asp, as well as an oxyanionic hole formed by the amino groups Gln and Thr. This triad is a well-known structural feature. This sequence is identical to that of serine proteases [33]. 

**Figure 2:**
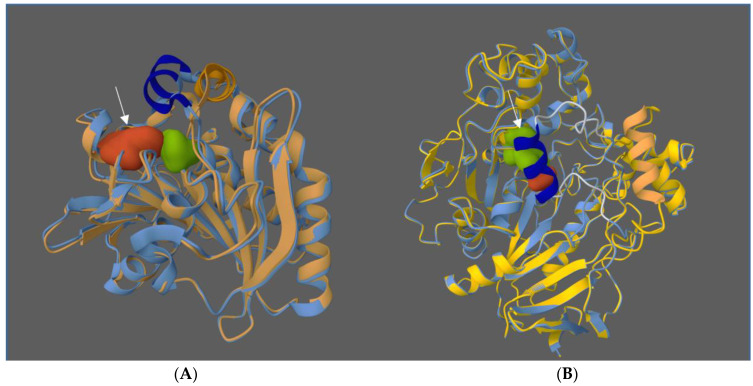
Structure of proteins made with the Protein Data Bank (PDB), with superposition of open and closed configuration of lipases from *Rhizomucor miehei* (**A**) and *Candida rugosa* (**B**). The active site is indicated by the arrow. Closed conformations are shown in blue. Shifting the cap (orange in the open conformation, deep blue in the closed), allows substrates access to the active site (sequence 4TGL-3TGL for *Rhizomucor miehei* [40,41] and 1CRL-1TRH for *Candida rugosa* [42,43]).

Pleiss et al. have studied eight different lipases and have made a precise description of them [37]. All of them can be described as having a large and hydrophobic catalytic site. Lipases can be classified into different groups according to the structure and localization in the protein of their active site [38]. Lipases such as *Rhizomucor miehei* have a hydrophobic active site located on the surface of the protein. *Candida rugosa* lipase forms a long tunnel (Figure 2). In addition, lipases generally have a cap, and the position of this domain determines the open or closed conformation of the enzyme [39]. ‘Cap’ refers to a domain of the lipase’s three-dimensional structure, linked to the rest of the protein by a flexible loop. In the closed conformation, the cap covers the active site, which is therefore unavailable to substrates. Conversely, in the open conformation, the cap does not obstruct access to the active site (Figure 2).

More precisely, if we look at the mode of action of lipases, the first step is the activation of the hydroxyl group of serine by a charge transfer relay concomitant a nucleophilic attack on the carbonyl atom of the substrate. To summarize, the formation of an acyl-enzyme intermediate results in the release of a leaving group, followed by nucleophilic attack on the acyl-enzyme intermediate by water from the microenvironment or from the external environment, leading to the formation of the product [44]. It should be added that this active site–substrate complex is stabilized by amino acids forming the oxyanion hole [45].The lipase-catalyzed reaction kinetics reflect a mechanism of the Ping-Pong Bi-Bi type [46]. 

Consequently, each lipase has its own structural characteristics. This singularity is due to the active sites of lipases and impacts chemoselectivity, regioselectivity, and even enantioselectivity [47]. For instance, Lipase B from *Candida antarctica* (CalB) is 105 times more selective for alcohol groups than for thiol groups, demonstrating an intrinsic ability to recognize different chemical groups [48]. Regioselectivity represents the preference of an enzyme for one atom rather than another from the same functional group, located in different positions in the substrate molecule. Two groups of lipases can be defined on their ability to distinguish between primary (sn-1,3) and secondary (sn-2) esters on a triglyceride. For example, the lipase from *Rhizomucor miehei* is sn-1,3 [49]. The regioselectivity of a lipase is reflected in the conformational way in which the substrate is bound to the active site [50]. 

It should be noted that the tertiary structure of lipases is dynamic and evolves according to its environment. For example, in non-aqueous environments, these proteins are more rigid. This reduction in conformational flexibility is due to the disulfide bonds and amino acid residues on the surface of the molecule. Water therefore plays a crucial role in enzymatic syntheses [51]. Lipases, which belong to the hydrolase enzyme class, use water for substrate degradation, but they can switch their activity from hydrolysis to esterification and transesterification. The direct environment of the enzyme, and therefore the reaction medium used, has an impact on protein structure and therefore on enzyme activity.

Table 1 allows us to appreciate the diversity of enzymes used according to carbohydrate substrate. As described in Table 1, lipases are extracted from different microorganisms. Lipase B from *Candida antarctica*, immobilized and commercially named Novozym435^®^, is the most referenced, and therefore employed, with its high activity, wide availability, and low price. From the point of view of SFAE formation, lipases are used to modify monosaccharides such as xylose, galactose, mannose, glucose, and fructose, but they are also prescribed for the esterification of disaccharides such as sucrose or lactose, and more rarely of more complex polymers [52]. Biomass can also be used. Recently, the synthesis of SFAEs catalyzed by CalB on xylose/glucose mixtures isolated from mixed hardwoods has been described [53].

### 2.2. Key Information

Lipases are active in reaction media composed of at least two distinct phases, in which all the reagents are distributed between these phases, even if their distribution is dynamic during the reaction [67]. Lipases have been described as active in a lot of solvents. Their native activity, i.e., the hydrolysis of triglycerides, is performed in an aqueous medium. It is possible to reverse this hydrolysis activity by choosing suitable organic solvents. Lipase-catalyzed esterification and transesterification reactions need a minimal amount of surrounding water in the enzymatic microenvironment, namely the amino acid triptyque Ser-His-Asp part of the protein structure [68]. The solvent can displace a water shell bound to the surface of the enzyme by hydrogen bonds, then causing a structural change in the enzyme [69]. The amount of water in the reaction medium, also called water activity, denoted as a_w_, is particularly important for lipase enzymatic activity and therefore in the observed rate of conversion of substrates to glycolipids. Water is present in the hydration layer of the enzymes, in the substrates, in the environmental humidity, or formed as a by-product during the reaction and may affect the thermodynamic and kinetic properties of the esterification reaction [70]. As mentioned above, lipases are more rigid in non-aqueous media, meaning that their mobility, relative to their active site, is lower [51]. It is also useful to clarify the crucial role of water in enzymatic synthesis. Water activity plays a key role in enantioselectivity, increased hydrophobicity decreases enantioselectivity, and different degrees of enantioselectivity can be achieved depending on the substrates used in enzymatic synthesis [71]. Reasoning about a_w_ allows us to characterize the reaction medium. When a_w_ is constant, enantioselectivity is better [72]. The three-dimensional structure of an enzyme is maintained by a complex equilibrium between hydrophobic interactions, electrostatic charge interactions, hydrogen bonds, disulfide bonds, and van der Waals interactions. Disruption of this balance and thus of these forces leads to protein unfolding. It has been established that lipase has greater activity in hydrophobic solvents than in hydrophilic ones [51]. Maintaining this active conformation, while avoiding switching to a hydrolase activity, is therefore going to be key to enzymatic synthesis.

The challenge when setting up a lipase reaction will be to find a solvent that possesses all these attributes. Moreover, Gonçalves et al. have carried out mapping of the parameters, in order to discriminate between them. They concluded that the solvent is the most studied parameter in the synthesis of SFAEs [73]. One of the crucial points to consider is the hydrophobicity of the solvents, usually classified through their LogP. It is the logarithm of the distribution coefficient of a substance in the water-octanol system, related to the availability of a substance in the different phases of a mixture. This value gives an indication of how hydrophilic or hydrophobic a solution is. Solvents with a logP < 2 can be considered polar; between 2 and 4, the polarity is intermediate; and a logP > 4 indicates a non-polar medium (Table 1). LogP and enzymatic activity can be correlated and used as partial prediction parameters. Solvents with a medium polarity, close to 1, are generally used, as they allow for sugar dissolution without interfering with enzymatic activity [74,75,76,77]. Solvents with a logP lower than 0 tend to remove water from the microenvironment and disperse the hydrophobic domains of the enzyme, thus inactivating it [78]. Maintaining the formation of the water layer surrounding the enzyme is important for stabilization, preventing enzyme aggregation [69]. In the reverse case, it has been observed that solvents with a logP close to 4, i.e., those that are non-polar, tend to reduce lipase flexibility [79]. Nevertheless, the literature sometimes reports a weak correlation between logP and thermodynamic parameters [70,80,81]. Many factors, such as substrates, must also be considered. For example, dipolar moment, hydrogen bonds, and polarizability also affect enzyme activity [51]. In a reaction to form a carbohydrate ester, the goal is to graft a lipophilic moiety (fatty acid) onto a hydrophilic molecule (sugar). In fact, the fatty acid is classically named the acyl donor whereas the sugar is the acyl acceptor. Under these conditions, it would therefore be better to use a solvent with a low logP, which dissolves both carbohydrates and lipids [82]. For example, dimethyl sulfoxide (DMSO), pyridine, or *N,N*-dimethylformamide (DMF) could be good candidates but are generally described as enzymatic inactivators, causing protein unfolding and thus lipase denaturation [83]. It should be highlighted that it is important to take into consideration the ease of removal of the solvents used and therefore the impact of these solvents in a global context. Solvents with a high boiling point are more difficult to evaporate and consequently require energy-intensive steps, going against green chemistry principles. Table 2 summarizes these different parameters that must be considered.

Many reaction media of varying degrees of complexity have been described in the literature for the synthesis of carbohydrate esters. Variability of media reaction is illustrated in Table 3. These systems are used to improve the enzymatic activity, especially to increase the solubility of the substrates at the initial time or to enhance the recovery of the reaction product. More specifically, Shin et al. show the close dependence between carbohydrate solubility and biocatalytic esterification rate [88]. In that sense, Degn et al. describe usable organic phases in single-phase systems [89]. Reyes and Duarte treat upon co-solvent systems; solvent-free systems are also well described [90], as are systems based on ionic liquids or supercritical CO_2_ [91].

Because the solubility of carbohydrates is dependent on the polarity of the solvent, tertiary alcohols are generally good candidates as reaction solvents for enzymatic synthesis [88]. Consequently, tert-butanol is the most used solvent (18%) in studies on enzymatically produced SFAEs, followed by 2-methyl-2-butanol (2M2B) with 12% occurrence [73]. Indeed, they are not substrates for lipases, do not cause any deactivation effects, and are easy to eliminate during purification steps [104]. Arcens et al. use acetonitrile to synthesize glucose palmitic ester. Their work is based on the low solubility of 6-*O*-glucose palmitate in acetonitrile, leading to its precipitation, and so the equilibrium is systematically oriented towards the formation of the desired product. Thus, a complete conversion in 40 h was obtained [98].

Co-solvent systems are widely studied in the literature [105]. Tertiary alcohols are usually combined with a solubilizing agent efficient enough to solubilize carbohydrates. Carbohydrate solubilization will lead to improved enzymatic stability and thus to a better selectivity [82]. It should be noted that the solubility of sugars can be overcome by using derivatized sugars, but the use of derivatized sugars increases the number of steps and the cost of synthesis [73]. Co-solvents are generally the preferable solution. Zhiwen et al. used tetrahydrofuran (THF) in tert-butanol, improving the water distribution in the system and reducing the unfavorable effect of THF, which naturally reduces enzyme activity (Table 2), by reducing water enrichment with THF’s hydrophobic character. During esterification reactions, water molecules are released and generally trapped by molecular sieves added at the beginning of the reaction. However, the captured water is also present in the microenvironment of the lipase, and thus the sieves can affect the synthesis of carbohydrate ester [81]. DMSO is also widely described as a co-solvent, allowing an increased carbohydrate solubility at the initial reaction time (Table 2) [106] and is mentioned in 5% of studies according to Gonçalves et al [73].

Immiscible co-solvents, such as acetonitrile/*n*-hexane mixture, are more rarely studied [100]. This bi-phasic innovative system allows for enzymatic synthesis in the acetonitrile phase and the extraction of reaction products from *n*-hexane in one step.

Green solvents are also used in biocatalysis. Broadly defined, there are 6 categories of green solvents. These are, naturally, water, supercritical fluids, fluorinated solvents, biobased solvents, and deep eutectic solvents (DES) [107,108]. Water cannot be considered here because carbohydrate esters cannot be formed. The greenest approach would be the solventless condition. In this sense, solvent-free systems are also employed and allow the decrease of reaction volumes and increase substrate concentration [109]. Hidayat et al. used a lipase immobilized on a hydrophobic matrix and a fluidized bed reactor, which minimized pressure compared with a packed bed reactor, to solubilize fructose and achieve solvent-free synthesis [92]. An other example is the synthesis of fructose oleic ester, achieved in a continuous system using *Rhizomucor miehei* lipase with 92% conversion rates, after 6 days’ reaction time [93]. The yields obtained are similar to those obtained with an organic solvent, but the reaction time is longer than with other systems [110]. Reaction time is not always increased in solvent-free synthesis. Other studies show an interest in solvent-free reactions, such as Aljawish’s team in the synthesis of formate ester [111]. The optimal conditions for formate ester synthesis were: 0.5 M of formic acid, 1.5 M of butan-1-ol in acetonitrile, with 2% of Novozym435^®^ at 40 °C and 400 rpm. Aljawish et al. synthetized these esters by reacting 1 M formic acid, 10 M butan-1-ol, and 2% Novozym435^®^ (*w*/*v*) at 40 °C and 400 rpm without molecular sieves in a solvent-free system. Under these conditions, using acetonitrile as a solvent led to an ester with a 90% yield in 8 h when using acetonitrile as solvent, and the same yield was obtained after 5 h in solvent-free conditions. The authors show that higher acid amounts lead to lower yields, hypothesizing a negative impact of the acid on the lipase. Thus, despite many advantages, especially regarding sustainable chemistry aspects, solvent-free systems are difficult to implement and depend extremely on reaction type.

At a higher level of complexity, some studies use innovative systems mixing supercritical CO_2_ and ionic liquids. Pure supercritical CO_2_ can dissolve small amounts of glucose. Tai and Brunner enhanced the bioavailability of their substrate by adding a highly polar organic solvent, such as acetone, which is tolerated in the food industry and in final products [91,112]. Thus, with 3% acetone, at 50 °C and 65 bar, they managed to make an innovative system with continuous esterification. In fact, with ionic liquid, syntheses are more efficient, due to an important bioavailability of the substrates at the beginning of the reaction. Systems using ionic liquids have been described recently in enzymatic systems and are a field in full expansion (12% of occurrences [73]). Abdulmalek et al. used a [Bmim][Bf4] (1-Butyl-3-methylimidazolium tetrafluoroborate) system with DMSO in 20:1 (*v*/*v*) ratio for the synthesis of galactose oleic ester surfactant. They obtained an 87% conversion of fatty acid after only a 2 h reaction time, with 2% (*w*/*w*) recombinant *Thermomyces lanuginosus* immobilized on silica (lipozyme TL IM^®^), at 60 °C, 300 rpm, and with a galactose/oleic acid ratio 1:3 [60]. Comparatively, Sabeder et al. obtained a 78% yield of 2M2B for the formation of palmitoyl-glucopyranose, after 72 h with Novozym435^®^ and 12.1% (*w*/*w*) molecular sieve at 60 °C, at 600 rpm [94]. The interest in ionic liquids is due to their reaction duration. Because ionic liquids are often described as toxic, other greener alternatives, such as DESs, are increasingly used. DESs are formed by mixing at least 2 compounds at an exact ratio corresponding to the eutectic point. Most of these solvents are liquid at room temperature, which facilitates their use [113]. Nevertheless, few studies currently report on DES–enzyme interactions [114]. Recently, the conformational stability of enzymes in DES has been highlighted [55]. Finally, syntheses using innovative biobased solvents such as 2-methyltetrahydrofuran-3-one (2MeTHF3one) or 2-methyltetrahydrofuran (2MeTHF) have been described recently [115]. 2MeTHF3one is a GRAS (generally recognized as safe) and food-grade solvent, and 2MeTHF is derived from furfural and levulinic acid. Vuillemin et al. compared these solvents to 2M2B for the synthesis of lauric glucose ester surfactant with lauric acid. 2MeTHF did not increase yields compared to 2M2B with similar molar yield (48%), whereas 2MeTHF3one provided a 79% yield. This study also shows increased enzymatic stability with 2MeTHF3one, measured with surface response design (PLS) [95]. Nowadays, other solvents used for biocatalyzed ester synthesis in general could also be used, such as methyl *tert*-butyl ether, cyclopentylmethylether, *p*-cymene, or anisole [116]. It is important to note that a solvent being biobased does not automatically make it eco-friendly [117]. It is important to consider how it is obtained and recycled. The choice is therefore complex and requires consideration of both enzymes and substrates, and also the facilities of post-synthesis treatments and the reaction duration. A summary of the advantages and limits of these various systems is presented in Table 4.

### 2.3. Temperature

Lipases are considered to be active between 40 °C and 80 °C. Nevertheless, thermal denaturation is observed beyond 60 °C [68]. Therefore, generally, lipases are used in temperatures below 60 °C. Lipases are thus thermosensitive, and their immobilization confers upon them a higher thermal stability. The temperature is a key factor. In fact, in most cases, it allows for the increase of substrates’ solubility. Conversely, the temperature reduction of the syntheses is a preoccupation of industry for environmental purposes. The literature allows us to appreciate the variability of optimal temperature used, generally between 40 °C and 60 °C [54,102,121]. For example, An et al. compared yields for the synthesis of *6-O*-(*N*-lauroyl-glycine)-d-glucopyranose at different temperatures [105]. They observed an increase in enzymatic activity resulting in yields increasing from 22% to 76% for temperatures of 40 °C and 55 °C, respectively, but a decrease when the reaction took place at 60 °C. This could be related to a thermal denaturation of the lipase at 60 °C. Nevertheless, it should be noted that some thermophilic enzymes, isolated from thermophilic microorganisms, show thermostability and activity at temperatures above 70 °C, such as lipase from *Thermomyces lanuginosus* [122,123]. Arcens et al. studied the effect of temperature on the synthesis of *6-O*-glucosyl palmitate surfactant in acetonitrile under inert atmosphere [98]. They showed that from 20 °C to 60 °C, the yields increased progressively, which allowed for a reduction in the reaction time, with all these parameters being linked to each other. A yield of 94% was obtained at 60 °C after 20 h reaction, while 40 h was necessary to obtain the same rate at 45 °C. At 70 °C, the enzymatic activity was not improved compared to 60 °C.

### 2.4. Substrate Molar Ratio

An acyl excess is generally desirable in order to promote a reaction, but it is also necessary to be careful of the substrate molar amount at the initial time. Indeed, too much carbohydrate could denature the enzyme by removing water from its microenvironment, preventing the active conformation of the protein [103]. The ratio employed will depend on the acyl chain length employed. Fatty acid chain length also influences enzymatic stability, the fatty acid itself having a LogP to be considered [124]. Lamsal et al. were interested in the synthesis of glucose ester and obtained interesting yields with a glucose–fatty acid ratio of 3:1 for palmitic, lauric, and hexanoic acids [97]. Conversely, Sebatini et al. were interested in 6-*O*-glucosyl stearate and obtained 87.2% yields from a 1:2 glucose–stearic acid ratio [125]. Optimizing the carbohydrate-to-fatty acid ratio is therefore largely dependent on the type of acid but also on the solvent used, as described in the previous section. It should be noted that the substrate molar ratio impacts the reaction medium, and consequently carbohydrate/ester solubility and enzymatic activity, causing a reduction in ester synthesis.

The type of acyl donor will also influence the reaction yields: when using transesterification, the problems of solubility are less important. It is thus possible to reduce the solvent volume and the reaction time. Vinyl esters as acyl donors are more frequently listed in the literature [73], since they are more reactive than the corresponding fatty acids, which generate water, which is more difficult to control [126]. Lin et al. synthesized a glucose lauric ester surfactant with *Aspergillus niger* lipase in 2M2B, using a vinyl laurate/glucose at a ratio of 2:1, in 5 h at 60 °C, with 50.9% yields [61].

There are therefore multiple factors to consider when setting up an enzymatic synthesis. Numerous researchers have enhanced the possible choices, in particular concerning solvents and biobased solvents. Understanding how lipase works is the key to enhance synthesis optimization. Nevertheless, it is necessary to keep in mind that all factors (i.e., lipase, reaction medium, temperature, acyl donor, acyl acceptor) are linked to each other. For example, a solvent will be more suitable according to appropriate corresponding conditions.

## 3. Latest Improvements and Recent Trends of the 21st Century

Biocatalysis, in the widest sense of the term, has become a major field of modern organic synthesis, particularly with protein engineering and advances in sequencing. This makes it a particularly cross-disciplinary field, without borders, involving multiple research units. That is why there are so many biocatalysis projects. The following section reviews the latest advances in enzyme-catalyzed reactions, and a focus is placed on lipase.

### 3.1. Recent Developments in Support Immobilization

As previously stated, lipase activity and stability strongly depend on temperature [68]. In addition to thermosensibility, reaction medium stirring, required to favor the formation of protein–substrate complexes, can also, progressively, denature the protein. Lipase immobilization on solid support can be a strategy to prevent this degradation. In fact, free enzymes are generally solid powders obtained by lyophilization or spray-drying, or even concentrated liquid solutions, and are difficult to manipulate, especially in recycling approaches. There is not a universal enzyme immobilization method, but an ideal approach requires a favorable interaction between the enzyme and its support. These interactions should provide a high surface area for exchange and display chemical, mechanical, and thermal stability, resistance to microbial degradation, as well as ease of regeneration. Meeting all these requirements is difficult, almost impossible: hence the notion of compromise. In addition, carrier properties need to be considered from the perspective of the potential application of immobilized enzymes. Immobilization will also confer a fundamental characteristic to enzymes, namely its reusability [6]. These reasons justify studies of immobilization supports different from those proposed by companies supplying them.

The size of pores, specific surface area, the immobilization method, and other parameters will play a role. Consider the most common enzyme, Novozym435^®^, trade name for CalB, produced by Novozymes: its immobilization is realized via interfacial activation on a resin, Lewatit VP061600. This resin is a macroporous support made of poly(methylmethacrylate) cross-linked with divinylbenzene. The particle size of the support is between 0.3 and 0.9 mm in diameter, with an overall enzyme loading of 20% (*w*/*w*) and 1 to 2% water bound to the protein [33]. Nevertheless, and despite quality support, the immobilized Novozym435^®^ has a decrease in activity from the 5th cycle of use, more or less important depending on the conditions. Some studies are interested in the reuse of enzymes, such as for example in the synthesis of *O*-(*N*-capryl glycine)-glucopyranose; the experimental conditions were 7% (*w*/*v*) supported by Novozym435^®^, molar ratio 1:1.5 for d-glucose-*N*-fatty acid glycine, at 55 °C. The biocatalyst was washed with *n*-hexane after each reaction. The first cycle yielded 78.6%, and the following cycle yields gradually decreased from 71.9%–70.3%–64.5% to 43.7%. This decrease in activity at the 5th cycle showed a gradual collapse of the enzyme activity before a more significant drop [105].

Interested in the CalB support, Cao et al. compared the yields obtained for Novozym435^®^, and CalB sp525, a commercial free lipase from *Candida antarctica* produced by Novozymes, immobilized on different supports by absorption and covalently grafted. They compared the activity of their supported biocatalyst in the synthesis of 6-*O*-palmitoyl glucose surfactant, compared to that of CalB sp435 in the same conditions. With CalB immobilized on polypropylene, they obtained 84% conversion, whereas the commercial CalB sp435 led only to 41% conversion after a 24 h reaction time. This shows a relative influence of the immobilization support, although each synthesis is unique. The authors concluded that the hydrolytic activity is totally independent of the water-absorption capacity, called aquaphilicity, but the conversion in the synthesis of 6-*O*-glucose palmitate increased with decreasing aquaphilicity of the support. Indeed, the less the affinity for water of the support, the more important the enzyme activity will be, an unfavorable sharing of water being possible in case of a hydrophilic support, eliminating water molecules around the lipases [124]. A hydrophobic support allows for an increased affinity for fatty acids and thus a better enzymatic activity.

Thus, immobilization has long been developed to provide a finer and higher degree of optimization to each synthesis [127].Another aspect that has become particularly important in recent years is the biobased quality of the immobilization carrier. Many green supports are now being investigated as alternatives to conventional materials. Many polymers can be used, such as alginate, chitosan, cellulose, agarose, guar gum, agar, carrageenan, gelatin, dextran, xanthan, and pectin. All these biosourced polymers are potentially good carriers. For example, Manoel et al. used an octyl agarose support to leave the lipase in a permanent open conformation, in contrast to covalent bond systems, which allow the lipase to open and close cycles [128]. By locking the lipase in the open position, they hoped to improve yields by facilitating and accelerating access to the active site. As another example, chitosan has different advantages compared to conventionally used supports: low cost, abundant, biodegradable, non-toxic, and highly adhesive. However, the use of biosourced polymers requires an increased vigilance in terms of microbial development. Recently, *Rhizopus oryzae* lipase was immobilized on rice husk, a by-product of the global rice industry. A comparison with the same enzyme immobilized on a commercial support has shown equivalent biocatalytic performances whatever the support was, but the recycling was more difficult for the enzyme immobilized on the rice by-product, due to greater fragility [129]. Green coconut fiber was also used to support CalB by physical absorption. Thermal stability was thus decreased between 50 °C and 60 °C compared to the unsupported enzyme. At 50 °C and 60 °C, the supported enzyme was 2 and 92 times more stable than the soluble enzyme, respectively. However, the stability of Novozym435^®^ at 60 °C was superior compared to the newly immobilized enzyme. In fact, after 10 h of incubation at 60 °C, Novozym435^®^ retained more than 70% of its initial activity, whereas the lipase immobilized on green coconut fiber had retained only 50% of its initial activity [130].

The amount of immobilized enzymes used can also have an impact. Indeed, the rate of esterification generally increases with the increase of the lipase load. For example, for the synthesis of *6-O*-(*N*-lauroyl glycine)-d-glucopyranose, An et al. improved their yield by increasing the amount of Novozym435^®^ employed from 1% to 7% (wt.%) [105]. The yields decreased slightly at higher lipase concentrations of 9%. Lower activity may result from the impossibility of reaching enzymatic reaction rates due to a low substrate availability [105].

Early examples of support-based optimization generally show poorer results than those obtained with Novozym435^®^. Nevertheless, the multiplicity of work on plant biomasses and nanomaterials will offer new opportunities in the choice of supports. A recent illustration is the use of 3D printing with, for example, carbon fiber-reinforced polylactic acid as enzyme support [131]. Innovative work has also demonstrated the close link between support and recycling. For example, magnetic supports facilitate lipase recovery and recycling. Examples of immobilization of magnetic particles, such as magnetic cellulose, enabling recovery of magnetic filters, have already been described [132,133]. These hybrid materials could be of interest in interdisciplinary and intradisciplinary fields and thus revolutionize a catalysis in constant evolution.

### 3.2. Potentiality of Multi-Enzymes

In addition to support aspects, new ways of synthesis using enzymatic cascades, e.g., in multi-steps syntheses or reactional sequences, are emerging to produce surfactants. The objective is to get rid of superfluous processing steps of linear syntheses to mimic as well as possible the complex metabolic pathways that cellular machinery can perform. Two approaches may be considered. The first is to employ enzymes in a multi-enzyme system, using multiple different immobilization carriers at once, and the second is to use a single carrier. This interest opens the way to an optimization of the support, and the challenge then becomes important. It is then possible to realize co-immobilization by conception of support bearing different enzymes, using random co-immobilization (on graphene, silica, polymers, carbon nanotube, or metal–organic frameworks) or compartmentalization, imitating the natural organization of enzymes in cellular environments [134]. It is possible to immobilize enzymes on themselves too, a phenomenon named cross-linked enzyme aggregates [135], realized by aggregation with ammonium sulfate further cross-linked with glutaraldehyde. Studies have already detailed multienzyme immobilization [134].

Multi-enzyme supports open the way to cocktail enzymes, generally used for the deconstruction of biomass, i.e., the hydrolysis of specific bonds, to obtain smaller fractions, more easily solubilized and applicable [136]. This interest in destructuring biomass is intricately linked to the economic crisis, particularly the oil crisis in 2022 [137]. Few studies relate the synergistic effect and combined use of enzymes, mentioning a lipase with a specific activity. Nevertheless, there could be an advantage to working with multi-enzymes, provided that the support is similar. This would complicate the reaction medium and consequently the treatment steps, contrary to green chemistry processes. It is possible to draw inspiration from the work conducted on biorefinery in order to feed the potentialities of enzymatic chemistry into the concept [107]. Articles dealing with this idea of cocktails, especially for hydrolysis activities on polymers, show the need to have several activities. By combining the effects of different enzymes, it is then possible to reduce time and enzyme dosage [138]. Huang et al. used a mixture of 3% Novozym435^®^ and 8% lipozyme TL IM^®^ for the transesterification of fat with methanol in tert-butanol [139]. The combined action of the two lipases allowed for the maintenance of constant enzymatic activity over 20 cycles, showing a potential ability to preserve the enzymes. It is possible to determine a synergistic effect yield by calculating the ratio of the yield obtained with the enzyme mixture to the yield obtained with a single enzyme [138].

### 3.3. Chemo-Enzymatic Synthesis

Mixed chemoenzymatic multi-step syntheses are also increasingly used. These mixed syntheses allow for the rapid improvement of industrial processes, in terms of yield and selectivity, but also according to green chemistry [140]. The term ‘hybrid catalysis’ is often used [141]. Buzatu et al. synthesized acetal derivatives of glucose, sucrose, and lactose by chemical means and then performed enzymatic esterification using Novozym435^®^ with 3(4-hydroxyphenyl)propionic acid [142]. Hybrid catalysis also makes it possible to get rid of the carbohydrate solubility problems. Indeed, it is possible to chemically modify glucose to obtain a less polar derivative, and thus solubilize it. This will limit bioavailability issues in the subsequent steps [142]. Sangiorgo et al. have thus derivatized glucose with 1-butanol using an Amberlyst 15 acid catalyst, and then conducted an esterification by Novozym435^®^ with lauric acid [143]. To go even further, Heuson et al. have coupled several types of chemical and biological catalysts with hybrid materials, allowing for co-immobilization. The support must then have multiple qualities. Synergistic effects allow yields and enantiomeric excesses rarely accessible by classical biocatalysis to be reached [144].

Another example with lipase is Villiger’s chemoenzymatic Baeyer oxidation with CalB, immobilized by adsorption on multiwall carbon nanotubes packed in a column reactor, at 40 °C. The biocatalyst allowed for the generation of peracids in situ, thus avoiding the need to manage these chemicals. In the case of the oxidation of 2-methylcyclohexanone to 6-methyl-ε-caprolactone, an 87% yield was obtained with a selectivity higher than 99%, in only 5 min of reaction time. In addition, high operational stability was observed, over more than 8 h of operation [145]. The combination of enzymatic synthesis and chemical synthesis enables the production of increasingly complex molecules.

### 3.4. Interest of Flow Chemistry

Flow chemistry is well discussed in the literature [127]. It corresponds to what is called continuous chemistry, as opposed to batch chemistry. The concept of flow chemistry revolves around the continuous pumping of fluids through a reactor to produce the product. More specifically, flow chemistry allows for an intensification of transfers, e.g., in a limited piece of equipment, to improve synthesis catalytic constants. Large-scale production and manufacture are based on the use of flow column reactors. Flow chemistry is ideally suited for lipases, especially because these biocatalysts do not require O_2_. However, few published data are available on the synthesis of SFAEs in flow chemistry conditions. Some studies, such as by Ruela et al. [146,147], have detailed the integration of immobilized enzyme systems and continuous flow reactors for the enzymatic esterification of glucose. In their work, they used a protected glucose (glucose ketal), immobilized lipase from *Rhizomucor miehei*, under discontinuous and continuous flow conditions and several fatty acids. They obtained high conversion rates in a short time. Flow microreactors for surfactant production are also described by Du and Luo. They used lipozyme TL IM^®^ absorbed on silica particles and obtained high yields for the synthesis of 6-*O*-palmitoylglucose (95%) in only 30 min [148]. This field has a strong potential for improvement in the coming years, especially if we combine all the possible advances, such as combining several enzymes or considering nanoarchitectonics, which enables the use of innovative strategies for integrating enzymes into continuous flow systems [149].

Concerning lipase-catalyzed reactions, other examples of flow chemistry are described in the literature. For instance, the synthesis of (±)-propan-1,2-diol with Novozym435^®^ was studied in a continuous flow reactor. Combining lipase and flow chemistry technology allowed the reaction to be reduced in time, from 6 h in batch to 7 min, keeping the conversion (up to 50%) and selectivity values high during the reaction procedure using methyl tert-butyl ether as solvent. Furthermore, when cascading enzymes are used, the ability to contain individual enzymes through immobilization in specific compartments can improve overall kinetics by providing reduced and more efficient pathways, avoiding undesirable cross-reactions, and facilitating cofactor regeneration [150]. Until now, there has been insufficient data on flow biocatalysis and the use of different biocatalysts in a simultaneous manner [151].

### 3.5. Molecular Bio-Imprinting as a Promising Tool for Simple Improvement

Molecular bio-imprinting is an interesting tool with lipases. It can be defined as an easy, durable, and inexpensive technique, based on imprinting of substrates on the active site of lipases. The conformation of the enzymes becomes more selective and stable, thus generating better catalytic performance. To our knowledge, this tool has not yet been used directly for the synthesis of SFAEs, but it has been described for lipases. Bio-imprinting was evaluated for *Burkholderia cepacia* lipase and porcine pancreatic lipase with lauric, myristic, palmitic, and stearic acids by Brandão et al. [152]. In this case, bio-imprinting improved the relative enzymatic activity 70 times for this enzyme. In particular, the effect was interesting for lauric acid and diminished when the chain was longer. FT-IR monitoring showed a decrease in α-helices and an increase in the β-helices of the enzyme, compared to Protein Data Bank data, translating to a greater protein rigidity and therefore an increased activity [152]. Bio-imprinting has also been reported in other studies in non-aqueous media [153]. This has only been shown for esterification reactions. The principle relies on a learning of molecular anchoring. Here, 57.34 mg of lipase was incubated in a 1:15 *v*/*v* fatty acid/isopropanol mixture, at 200 rpm and 25 °C, thus assumed to be non-denaturing, for 60 min. The fatty acid was removed with 20 mL octane, and the solids were recovered. The effect of bio-imprinting can be monitored by FT-IR analysis of lipase structures [152,154]. Bio-imprinting could for example prove its worth in cases of multi-step synthesis. Indeed, the nature of the leaving group, i.e., the alkyl chain of the acyl donor, involved in the first step, would influence reaction enantioselectivity.

### 3.6. Enzyme Engineering

Although nature offers us a great diversity of enzymes and consequently of activities, syntheses carried out by natural enzymes are limited in effective substrates and types of reaction, which limits their application in chemistry. A biocatalyst, for instance, needs to be robust to be competitive [155]. Accordingly, chemists often want to modify and adapt enzymes by introducing transformations that do not occur in nature [156]. A distinction can be made between commercial and non-commercial enzymes, generally referred to as wild type. The commercial enzymes available are generally purified enzymes, used in the laboratory and the pharmacy, whether immobilized or not, or lyophilized cell lysates, which are consequently more complex. Concentrated solutions obtained from fermentation systems are also used in the food industry. To obtain enzymes that are more specific to a reaction, a substrate, or a parameter, it is possible to bypass the commercial enzymes. To achieve this, it is necessary to find the exact enzyme corresponding to the desired criteria. Databases list current research on available enzymes [157]. For example, SciFinder, Uniprot and BioCatNet are databases that can be used to identify enzymes able to catalyze the desired chemical transformations [158]. These sites are virtual libraries, referencing the metagenomic data accumulated on biocatalysts [159]. These tools allow researchers to choose an enzyme by discriminating requests according to criteria.

**Figure 3:**
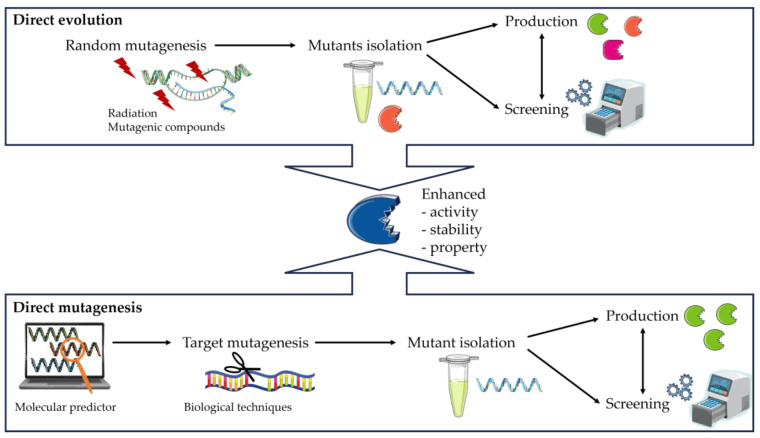
Schematic illustration of two different strategies, direct evolution, and direct mutagenesis, used for the modification of enzyme.

Moreover, wild type enzymes, purified from microorganisms, cannot generally be used in organic synthesis, particularly on an industrial scale [160]. To make these enzymes usable, they need to be modified to enhance certain properties. These changes could, for example, modify the specificity, the substrate selectivity, the efficiency, or the catalytic stability. Artificially modifying an enzyme is an alternative to searching for and finding new lipases in nature [161]. One technique used to make these enzymes more easily applicable to chemistry is ‘directed evolution’, which can be contrasted with ‘directed mutagenesis’ [162]. It corresponds to the 3rd wave of biocatalysis, and the objective is to design mutants of existing lipases [163]. The objective is to design mutants of existing lipases. To design these enzymes, we need to distinguish between the random and the rational approaches, which requires extensive structural knowledge of the protein (Figure 3). Directed evolution is a random approach, aimed at obtaining a protein by imitating the process of natural selection, in order to ‘direct’ evolution. These approaches are described in particular by Damborsky and Brezovsky, Dorn et al., and Chen and Arnold [164,165,166]. The directed evolution cycle involves repeated cycles of design to generate DNA libraries, using conditions approaching natural ones such as radiation or mutagenic compounds, and then screening the resulting proteins. Several properties can be optimized in parallel, and then the proteins are isolated and characterized. To sum up, there are 3 main stages in directed evolution:

- The creation of a library of DNA variants using molecular biology techniques is the first stage.
- The target proteins are then produced using host cells.
- Finally, the enzymes are evaluated using functional screening techniques such as assays to assess the effects of the modifications, such as increased selectivity, reaction specificity, or solvent resistance.

The final stage involves the complete isolation and characterization of the most interesting protein clones (Figure 3). In contrast, direct mutagenesis is site-specific. It is used to make specific and intentional mutant changes to the DNA sequence of a gene or gene product. Biological techniques such as clustered regularly interspaced short palindromic repeat (CRISPR) can be used but are highly complex [167].

The work presented here focuses specifically on lipase modification. The efficiency of lipases is generally attributed to their active sites. The complexity of the active sites, designed by nature, makes it possible to position the catalytic residues precisely to stabilize the transition state, and therefore perfectly optimize the conditions for a given reaction [168]. These active site residues are generally described in lipase-modification studies. Targeted mutations in the active site can enable remodeling of the binding to the substrate and thus potentially obtain activity towards substrates. The development of modified lipases (mutant) with new properties is possible thanks to increasing knowledge of three-dimensional structures in parallel with advances in bioengineering [169]. In addition to the active site, there is an inherent flexibility in the enzyme structure. Mutations at the protein surface and in the flexible regions of the structure can often lead to improved solvent tolerance and thermostability. It is in this sense that enzymes can adopt different conformations, such as open and closed for lipases, which constitutes a substrate binding and product release step [170]. This understanding of the phenomenon is made possible by advances in enzyme topography [171].

Theoretically, all modifications are possible by mutagenesis [172]. Concerning lipase, the catalytic activity could be improved by mutation of the catalytic site of the oxyanion hole [47]. Indeed, studies have shown that mutation on oxyanion residues can affect the characteristics of lipases. Several examples can be found in the literature. Wahab et al. worked on the *Geobacillus zalihae* lipase, which has 2 oxyanion residues, Q114 and F16, a glutamine and a phenylalanine, respectively. The specific change of this glutamine residue to phenylalanine enabled the researchers to obtain a mutant of *Geobacillus zalihae* lipase, which has better properties, such as increased optimal temperature and increased stability in organic solvents [173]. Another example concerns *Yarrowia lipolytica* lipase. Kumari’s team showed that an F146L mutation on the phenylalanine residue led to changes in substrate binding and consequently to an increase in catalytic efficiency [174]. Similarly, the F17S mutation in *Bacillus thermocatenulatus* lipase allowed for an increase in the enzymatic activity of this lipase in organic solvents [175]. A mutation in the histidine amino acid (H110) in *Pseudomonas statzeri* lipase gave a mutant H110F lipase, which has an enzymatic activity 4 times greater than that of wild type lipase. Ma’ruf et al. sought to understand the impact of a mutation in this H110F residue using LK4 lipase as a model [176]. Mutation of this histidine residue enabled the enzyme to become active in a solvent such as *n*-hexane, contrary to the wild type enzyme. In addition, this single mutation resulted in a higher affinity of this lipase for certain substrates such as palmitic acid [176]. This work shows that it is not necessary to make significant changes in a protein sequence to obtain an interesting mutant.

The selection of the mutant of interest is a long and difficult task. The exponential increase in knowledge about enzymes, combined with technological and biological advances, means that complex genomic and proteomic databases can be created. This data can then be processed by artificial intelligence (AI), with predictive functional analysis, imposing discriminating criteria, to perform screening [177,178]. Simply considering machine learning, which is a subset of AI, 2 approaches are possible for predicting a synthesis [179]: non-supervised learning and supervised one. The second approach is favored in enzymology [180], since 3 steps are required to implement this supervised learning [181,182]: (i) data collection from databases (brenda, enzymeML, PDB, UniprotKB), (ii) data processing by an algorithm to select data and (iii) validation of the computer model with a mix of data sets, to test robustness [180]. The aim is to find enzyme variants with the enantioselectivity required for a reaction [183].

For example, Yoshida et al. screened around 8000 variants for a *Burkholderia cepaia* lipase mutant with improved thermostability obtained through directed evolution [184]. The authors used high-throughput screening techniques. Using genetic and protein data from around 200 selected mutants, the analysis and use of AI with machine learning (computer-generated screening) enabled the analysis to be reduced to 20 candidates based on a ‘residue/physico-chemical properties’ relationship. Among 20 lipases tested, a triple mutant emerged with increased initial and residual activity at 60 °C compared to the wild type and the other tested candidate lipases [184].

Advances in informatics have also enabled the development of predictive synthesis systems. Scientists usually work from a target molecule or a specific substrate to develop a biocatalytic process in one or more stages. This process is traditionally carried out by ‘manual’ design, using the expertise of the scientists or the existing literature. Predictive systems, which have appeared very recently, are not intended as predictors of enzymatic activity, but predict the outcome of a reaction based on databases. Recently, programs such as RetroBioCat, which are free toolbox enzymes, have been created to help researchers with their development [185]. They are particularly useful when you want to set up experiments with atypical substrates [186]. They are also used to predict the stereoselectivity of a system. An example of these predictive tools is the system developed by Probst and his team. They predict reactions with ‘molecular transformer trained enzyme-catalyzed reactions’. The main actual drawback is the lack of available data in the databases, particularly for certain enzyme classes. In fact, larger quantities of data mean greater accuracy, with a correlation of 60% advanced by Probst et al. [186]. Advances in protein engineering and AI therefore open up a whole host of possibilities for obtaining SFAEs. 

The list of abbreviations used in this article is included in Table 5.

## 4. Conclusions

The parameters presented in this review seemed relevant in SFAE enzymatic synthesis. One of the advantages of the use of enzymes is the potentiality of this tool, and thus the possibility of optimizing seemingly unlimited synthesis. Enzyme selection is the central point around which all the other parameters are designed. The understanding of the action mechanisms of the lipase and consequently the inter-enzymatic variability should be one of the first parameters to be considered. The selection of the reaction medium has been identified as a crucial aspect. Indeed, the polarity of the reaction medium influences the solubility of substrates, and therefore their bioavailability. And in turn, this has an impact on biocatalyst behavior, and therefore on reaction kinetics. This review highlights, using several references, that a long reaction time does not necessarily lead to higher yields, nor does a large amount of lipase. In fact, parameters are all related to each other. Then, a focus was placed on recent advances in biocatalysis, such as the possibilities that bio-imprinting will offer to researchers in the coming years. Particular attention was paid to enzyme carriers, cascade synthesis, and multi-enzyme synthesis, which are certainly the key to producing enzymatically complex surfactant molecules. Chemo-enzymatics is also outlined as a field that would allow us to sustainably carry out an infinite number of already existing syntheses. Chemo-enzymatic synthesis, flow chemistry, and multi-enzyme cascade reactions have been, are, and will continue to be major topics of interest. Flow chemistry is particularly used in industry, with flow column reactors, and is a major contributor to scaling up and technology transfer between academics and industrials. A strong demand for personalized enzymes is also emerging. Indeed, genetic engineering could allow us to design lipases on demand in the coming years and thus increase the fields of applications, improving efficiency, enabled by integrating AI into biotechnology processes. This review gives the keys for tomorrow’s biocatalysis, highlighting the importance of tailoring enzymes to specific reactions and processes.

## Figures and Tables

**Table 1:** Diversity of lipase used for the biocatalytic synthesis of amphiphilic carbohydrates in recent studies.

Acyl Acceptor	Enzyme	Acyl Donor	Product	Solvent	Ref.
d-Allose	Novozym435^®^ (*Candida antarctica* immobilized lipase B)	Vinyl caprylate	1,6-diacyl-d-psicofuranoses	Acetone, acetonitrile, or tetrahydrofuran (THF)	[54]
d-Arabitol	Novozym435^®^	Lauric acid	1,5-dilauryl-d-arabitol	Reactive natural deep eutetic solvent (R-NADES) ChCl:d-arabitol	[55]
d-Fructose	Immobilized *Rhizomucor* *miehei* lipase	Oleic acid	Fructose oleate *	2-methyl-2-butanol (2M2B)	[56]
	Novozym435^®^	Lauric acid	6-*O*-lauroyl-d-fructofuranose	Ethyl-methyl ketone	[57]
	Novozym435^®^	Myristic acid	d-fructosyl myristate *	Tert-butanol (tert-BuOH):pyridine (11:9, *v*/*v*)	[58]
d-Fructose	Immobilized *Rhizomucor* *miehei* lipase	Myristic acid	Fructose myristate *	Solvent-free	[59]
d-Galactose	Immobilized *Rhizomucor miehei* lipase	Oleic acid	Galactose oleate *	[Bmim][BF_4_]/dimethyl sulfoxide (DMSO) 1-Butyl-3-methylimidazolium tetrafluoroborate	[60]
d-Glucose	*Aspergillus niger* lipase	Lauric acid	*6-O*-lauroyl-d-glucopyranose	2M2B	[61]
	*Aspergillus oryzae* lipase				
	Lipozyme *TL IM^®^* (*Thermomyces lanuginosus* immobilized)				
	Novozym435^®^				
	Novozym435^®^	Myristic acid	Glucosylmyristate *	2M2B	[62]
d-Maltose	Novozym435^®^	Ethyl Butanoate	6-*O*-butyrylmaltose	tert-BuOH	[63]
Lactose	*Candida antarctica* lipase (Novozym435^®^ and immobilized on Immobead)	Lauric acid	Lactose monolaurate *	Acetone	[64]
Lactose	Free lipase (MAK Wood)	Lauric acid	Lactose monolaurate *	Acetone	[64]
L-Rhamnose	Free *Pseudomonas stutzeri* lipase	Lauric acid	4-*O*-lauroyl-rhamnose	Anhydrous THF	[65]
d-Maltotriose	*Thermomyces lanuginosus* lipase (immobilized on celite and granulated with silica (Lipozyme TL IM^®^))	Vinyl laurate	6-*O*-lauroyl-maltotriose	2M2B/DMSO (5 or 20% *v*/*v*)	[66]

* Binding site has not been specified.

**Table 2:** Enzyme activity, glucose solubility, logP, and boiling point of different solvents.

Solvent	Novozym435^®^ Activity at 45 °C (µmol·min^−1^·g^−1^)	Glucose Solubility at 45 °C after 24 h Incubation (mM)	LogP	Boiling Point (°C)	References
*N,N*-dimethylformamide (DMF)	0	12	−1.0	153	[81,84,85]
DMSO	0	29	−1.35	189	[81,84,85]
*n*-hexane	0	0	3.9	69	[81,84,86]
THF	1.6	2.1	0.46	65	[81,84,86]
tert-BuOH	3.7	12	0.35	82	[81,84,86]
2M2B	3.6	10	0.89	102	[81,84,86]
Pyridine	0	134	0.65	115	[81,84,87]

**Table 3:** Different solvents influencing the synthesis of amphiphilic carbohydrates (atm means atmospheric pressure). Yield values are indicated by *.

Saccharide (Carbohydrate)	Acyl Donor	Enzyme	Solvents (*v*/*v*)	Time Temperature Pressure	Conversion of Fatty Acyl Donor or Yield *	Ref.
d-Allose	Vinyl esters	Novozym435^®^	Acetonitrile	24 h 45 °C atm	83% *	[54]
d-Arabitol	Lauric acid	Novozym435^®^	R-NADES (reactive natural deep eutetic solvent) ChCl:d-Arabitol	24 h 70 °C atm	95% *	[55]
d-Fructose	Lauric acid	Novozym435^®^	[Bmim][TFO] */2M2B (3:2) * 1-Butyl-3-methylimidazolium/trifluoromethanesulfonate	12 h 50 °C atm	85% *	[57]
	Oleic acid	Immobilized *Candida rugosa* lipase	Solvent-free	48 h 60 °C atm	80% *	[92]
	Oleic acid	Immobilized *Rhizomucor miehei* lipase	Solvent-free	144 h 65 °C atm	92%	[93]
	Palmitic acid	Novozym435^®^	2M2B	72 h 40 °C atm	78% *	[94]
d-Galactose	Oleic acid	Immobilized *Candida rugosa* lipase	DMSO/IL[Bmim][BF_4_] * (1:20)	2 h 60 °C atm	87%	[60]
d-Glucose	Lauric acid	Novozym435^®^	2MeTHF (2-methyltetrahydrofuran)	72 h 75 °C atm	48% *	[95]
			2MeTHF3one (2-methyltetrahydrofuran-3-one)		79% *	
	Ethyl laurate	Supported *Aspergillus niger* lipase	2M2B/2MeTHF3one (4:1)	48 h 56 °C atm	49% *	[96]
	Vinyl laurate				80% *	
	Lauric acid	Novozym435^®^	DMSO/tert-BuOH (4:1)	24 h 55 °C atm	77%	[97]
	Palmitic acid		Acetone saturated with supercritical CO_2_ in continuous reactor	4 h 50 °C 65 bar	>20%	[91]
	Vinyl palmitate		Acetonitrile	72 h 45 °C atm	100% *	[98]
d-Maltose	Lauric acid	Novozym435^®^	Acetone/*n*-hexane (3:2)	72 h 50 °C atm	69%	[82]
d-Mannose	Capric acid	Immobilized *Candida rugosa* lipase	Acetone	48 h 50 °C atm	62%	[99]
	Lauric acid	Novozym435^®^	*n*-hexane/acetone (1:1)	72 h 50 °C atm	25% *	[100]
	Vinyl myristate	Novozym435^®^	[Bmpyrr] */[TFO] * 1-butyl-1-methylpyrrolidinium	24 h 60 °C atm	71% *	[101]
d-Xylose	Vinyl laurate	Novozym435^®^	2M2B	4 h 60 °C atm	25%	[102]
	Hexanoic acid	Novozym435^®^	DMSO/Acetone (1:10)	24 h 60 °C Atm	64%	[103]

Note: Results are given in both conversion rate and yield. The conversion rate provides information regarding the proportion of reagent that has disappeared, generally the fatty acid, but not on the amount of product formed, which depends on selectivity.

**Table 4:** Presentation of the various chemical systems.

Reaction System	Advantages	Limits	References
Solvent	Easy to use and implement	Limitation of soluble substrates in a single solvent May affect enzyme activity Toxicity Environmental impact	[69,82,83,88,118]
Co-solvent	Combines the respective advantages of the two components Design flexibility: components and ratio	Need to handle and manage several solvents Toxicity Environmental impact	[105,119]
Solvent-free	Reduction in the cost and environmental impact Reduced reaction volume and increased substrate concentration Simplification of purification steps	Absence/limitation of substrate solubility Reduced homogenization Affected enzymatic activity Conditions of use may be incompatible with enzyme and substrates	[90,109]
Supercritical CO_2_/solvent	Combines the respective advantages of the two components Reduced toxicity	May require temperature and pressure conditions that are not compatible with the enzyme and substrates	[91,112]
Ionic liquids	Increased solubility of substrates Low volatility	Generally expensive Toxicity Environmental impact Conditions of use may be incompatible with the enzyme and substrates	[60,94,120]
DES	Increased solubility of substrates Low volatility Design flexibility: components and ratio More durable and environmentally friendly	Design complexity: combination and ratio of components Must be compatible with the enzyme and substrates and have the required properties (substrates solubility)	[113,114]

**Table 5:** List of abbreviations used in this article.

	Abbreviation	Full Name
Organic solvent	2M2B DMSO	2-methyl-2-butanol Dimethyl sulfoxide
	DMF	*N,N*-dimethylformamide
	THF	Tetrahydrofuran
	2MeTHF	2-methyltetrahydrofuran
	2MeTHF3one	2-methyltetrahydrofuran-3-one
	R-NADES	Reactive natural deep eutetic solvent
	ChCl	Choline chloride
	*tert*-BuOH	Tert-butanol
	[Bmim][BF_4_]	1-Butyl-3-methylimidazolium/tetrafluoroborate
	[Bmim][TFO]	1-Butyl-3-methylimidazolium/ trifluoromethanesulfonate
	[Bmpyrr]	1-butyl-1-methylpyrrolidinium
	IL	Ionic liquid
	DES	Deep eutetic solvent
Lipase	CalB	Lipase B from *Candida antarctica*
	TLL	Lipase from *Thermomyces lanuginosus*
Biocatalyst	Novozym435^®^	Lipase B from *Candida antarctica* produced by Novozymes
	Lipozyme TL IM^®^	Recombinant TLL immobilized on silica
